# Pegvisomant for acromegaly: does it always works?

**DOI:** 10.20945/2359-3997000000163

**Published:** 2019-08-14

**Authors:** Sebastian J. Neggers, Aart J. van der Lely

**Affiliations:** 1 Erasmus University Medical Center Division of Endocrinology Department of Medicine Erasmus University MC Rotterdam The Netherlands Division of Endocrinology, Department of Medicine, Erasmus University MC, Rotterdam, The Netherlands

Two studies published in this present issue of *the Archives of Endocrinology and Metabolism*, by Natalia Garcia Basavilbaso (1) and coworkers, as well as the one by Cesar Boguszewski and coworkers (2), describe the treatment response in real-life of pegvisomant (PEGV) therapy in acromegaly patients, including aspects of safety and efficacy.

Taken together, these data reflect the everyday practice results of 184 patients from 27 dedicated centers from Brazil and Argentina. These two essays are essential and very welcomed as they report indeed what is happening in real-life with a compound that, in theory, as well as during registration studies, somewhat promised to be efficacious in controlling disease activity in virtually all acromegaly patients (3,4). However, as was also reported in both older and newer publications from the analyses of data coming from the Pfizer Inc Initiated Global, non-interventional safety surveillance study of long-term treatment with PEGV (ACROSTUDY^tm^), the efficacy of PEGV in normalizing serum IGF-I levels apparently does not exceed about two-thirds of treated patients (5,6).

In the Argentine study, 43 patients (45%) received PEGV monotherapy, while 41 (55%) received combination therapy (68.3% with long-acting somatostatin analogs). Serum IGF-I levels decreased to normal ranges in “only” 63% of patients after a median treatment duration of 27 months, with a daily mean dose of about 12 mg (2). In the Brazilian report, PEGV was used as monotherapy in 11% of the cases, while normal IGF-I levels were obtained in about 75% of patients (1). Why more patients cannot be controlled in the real-life setting of center of excellence? This still remains a very significant question. A very important issue, for sure, is that in none of the published reports on long-term efficacy data, the maximal allowed dose of PEGV (30 mg daily) has been used in those subjects that were not well controlled in their IGF-I levels. Also, in both reports in this issue of the Journal, it was observed that the mean and maximal dosages of PEGV could have been higher, especially in non-controlled subjects. Data from Brazil, e.g., show that normalization of serum IGF-I levels at any point during therapy was obtained in 80 (74.1%) patients: 11 (92%) in monotherapy and 69 (71%) in combined treatment. The median maximum dose of PEGV in monotherapy was 15 mg/day and in combined treatment it was 10 mg/day, but these values appeared not to be statistically different. The Argentine data mentioned that the prescribed dose of PEGV ranged from 20 to 210 mg weekly, which was accompanied by a normalization of IGF-I levels in 53 % and 47% of combined and monotherapy treatment respectively, and as with the Brazilian data without a significant difference between those two. Noteworthy is that the group of patients in the Argentine study not achieving disease control, received an average daily dose of 13,9 mg that could have left room for an increase up to 30 mg daily. Although not allowed by the label, some patients need more PEGV to have their IGF-I levels normalized. That is another lesson that ACRODAT thaught us (7). Gathering all the literature data and the two studies in this issue, it appears that patients who need more PEGV to normalize their IGF-I levels have a more aggressive disease, as they are younger, have higher baseline IGF-I levels, more hypertension, more sleep apnea and diabetes and are more overweight. A better understanding of this dose-efficacy relationship of PEGV might avoid inappropriate dosing and prevent serum IGF-I levels from remaining unnecessarily uncontrolled. Both studies don’t observe much difference between the PEGV doses used in the controlled versus the uncontrolled groups, which is difficult to interpret since a proper dosing has not been used in either of the groups. Therefore, the major challenge for advocates of PEGV treatment in the coming years must be to instruct clinicians to use PEGV in doses that do normalize IGF-I is almost all subjects and don’t stop halfway in dose titration.

When safety is concerned, both studies address this very well and the data show that the well-known side-effects of localized lipodystrophy and hepatotoxicity do occur and the reported incidence varies with the interval between observation of the patients, mostly in an outdoor setting. Data from ACROSTUDY probably underscore the real incidence because of the wider interval between reported visits of 6 months to 1 year (5,6). However, all in all, these side effects appear to be not frequent or serious enough to prevent large-scale use of PEGV in acromegaly patients that really need such an effective drug as it remains by far the most effective medical treatment available to date. What remains important to follow up closely is the potential increase in tumor size in patients on PEGV treatment, especially in those without concomitant somatostatin analog treatment. Both studies mentioned this well and in detail. One could conclude that this does happen in some subjects with aggressive tumors and that none of the treating physicians link it to the use of PEGV, but the incidence is very low and well below 5%.

In conclusion, these two large retrospective studies from South America nicely add important real-life data to the increasing pile of reports on safety and efficacy of PGV in the treatment of acromegaly patients. Both again show that proper dosing of PEGV in all patients remains a challenge and, for sure, leave room for even higher efficacy rates in the future.
